# Comparison of fluorescence-based techniques for the quantification of particle-induced hydroxyl radicals

**DOI:** 10.1186/1743-8977-5-2

**Published:** 2008-02-28

**Authors:** Corey A Cohn, Sanford R Simon, Martin AA Schoonen

**Affiliations:** 1 Center for Environmental Molecular Science, Stony Brook University, Stony Brook, USA; 2 Department of Geosciences, Stony Brook University, Stony Brook, USA; 3 Department of Pathology, Stony Brook University Hospital, Stony Brook, USA; 4 The National Research Centre for the Working Environment, Copenhagen, Denmark

## Abstract

**Background:**

Reactive oxygen species including hydroxyl radicals can cause oxidative stress and mutations. Inhaled particulate matter can trigger formation of hydroxyl radicals, which have been implicated as one of the causes of particulate-induced lung disease. The extreme reactivity of hydroxyl radicals presents challenges to their detection and quantification. Here, three fluorescein derivatives [aminophenyl fluorescamine (APF), amplex ultrared, and dichlorofluorescein (DCFH)] and two radical species, proxyl fluorescamine and tempo-9-ac have been compared for their usefulness to measure hydroxyl radicals generated in two different systems: a solution containing ferrous iron and a suspension of pyrite particles.

**Results:**

APF, amplex ultrared, and DCFH react similarly to the presence of hydroxyl radicals. Proxyl fluorescamine and tempo-9-ac do not react with hydroxyl radicals directly, which reduces their sensitivity. Since both DCFH and amplex ultrared will react with reactive oxygen species other than hydroxyl radicals and another highly reactive species, peroxynitite, they lack specificity.

**Conclusion:**

The most useful probe evaluated here for hydroxyl radicals formed from cell-free particle suspensions is APF due to its sensitivity and selectivity.

## Background

The particle-induced formation of hydroxyl radicals has gained considerable attention by toxicologists, environmental scientists, and geochemists [1]. The hydroxyl radical is a molecule with an unpaired electron, which will react nonspecifically with most organic molecules within nanoseconds after their formation [2]. It is included in a group of molecules termed reactive oxygen species (ROS), which also includes hydrogen peroxide (H_2_O_2_) and superoxide (O_2_^•-^). Hydroxyl radicals (^•^OH) have been implicated in genotoxicity [3,4] and oxidative stress [4-6] that may contribute to lung disease upon exposure to asbestos [7], silica [3,8], and other airborne particulate matter [9,10]. Hence, ^•^OH formation *in vitro *and *in vivo *has been used as an indicator for particulate-induced toxicity potential [3-5,11-14].

The detection of hydroxyl radicals is not a trivial process; detection methods require high sensitivity, high selectivity, and low detection. The extreme reactivity of ^•^OH precludes its direct detection by conventional spectroscopic methods, and instead, techniques employ reaction of ^•^OH with a target molecule (*probe*). Upon reaction, the probe's characteristics such as light absorption, fluorescence, and/or electron spin resonance may change. These changes in the probe's characteristics can be correlated with estimated concentrations of ^•^OH (or another ROS) generated under controlled conditions. The resulting calibration curve can then be used to quantify particulate-generated ^•^OH.

Several techniques have been employed for detecting particle-induced formation of ^•^OH. Electron spin resonance (ESR) spectroscopy is a technique for detecting molecules with unpaired electrons. By adding a probe molecule such as 5,5-dimethyl, 1-pyrroline *N*-oxide (DMPO), commonly referred to as a *spin trap*, the lone electron on ^•^OH can be captured (trapped) on the DMPO molecule (i.e., DMPO-OH). The reaction product, DMPO-OH, is also a radical species, but it has a sufficiently long half life so that it is possible to record its distinctive electron paramagnetic resonance spectrum [15-18]. In other techniques involving cells or tissues, biological products of ^•^OH-induced oxidation which can be subsequently quantified include lipid hydroperoxides and aldehydes [19], DNA strand-breaks [3,20], products of RNA degradation [21], nucleotide base oxidation products [22-24] and multiple signaling molecules such as cytokines [25,26] and the transcription factor, p53 [27], the upregulation of which is indicative of inflammation and apoptosis (i.e., programmed cell death). Still other methods employ a probe molecule which may undergo changes in absorbance (e.g., leuco crystal violet) [28] or fluorescence [e.g., 2',7'-dichlorofluorescein (DCFH)] [29-31] when oxidized by hydroxyl radicals.

Although these techniques are suitable for certain experimental objectives, they all have limitations. Some techniques are better adapted to use with viable cells and others to cell-free solutions, some methods require specialized equipment and others, additional probe preparation steps. The electron spin resonance method with DMPO has been used extensively but it requires specialized equipment (i.e., an ESR spectrometer) and the method is susceptible to artifacts when ferric iron is present [e.g., Fe(III) associated with a particle] [32]. In addition, the requirement for tuning the ESR spectrometer and the short half-life of DMPO-OH (i.e., 2.9 minutes [33]) may lead to irreproducible results and difficulties in generating calibration curves. 2',7'-dichlorofluorescein (DCFH) has been used widely, but it will react with several reactive oxygen species, not just hydroxyl radicals [31]. Since the diacetate ester of DCFH (H_2_DCFDA), which is frequently employed because of its high permeability to viable cells, will not react with ^•^OH unless it undergoes a de-esterification step either within cells or chemically [30], its use with particle suspensions in the absence of cells requires an extra step in the method protocol. Evaluating the fate of nucleic acids after exposure to particles is another technique that has been employed for the detection of particle-generated hydroxyl radicals [3,20,21]. Although this method is useful for estimating a genotoxic potential from exposure to certain particles, it is not well adapted for quantifying hydroxyl radical concentration, especially when the multiple products of reaction with nucleic acid are formed *in situ *within viable cells. In addition, when cellular DNA degradation is evaluated, DNA strand repair mechanisms may reduce the level of particle ROS-induced strand breaks [34].

For this study, our objective was to evaluate several hydroxyl radical detection techniques that could overcome some of the challenges described in the preceding paragraph. Several relatively new compounds have been developed for ROS detection using fluorescence. Some of them will detect many ROS and others have been designed to react only with highly reactive oxygen species such as ^•^OH. Three of the five probes evaluated will react directly with ^•^OH, while the other two require reaction of ^•^OH with another molecule that can directly react with the probes.

### Probes selected for evaluation

Five probes were evaluated: 3'-(*p*-aminophenyl) fluorescein (APF), 10-acetyl-3,7-dihydroxyphenoxazine (amplex ultrared), 2',7'-dichlorodihydrofluorescein diacetate (H_2_DCFDA), 5-(2-carboxyphenyl)-5-hydroxy-1-((2,2,5,5-tetramethyl-1-oxypyrrolidin-3-yl)methyl)-3-phenyl-2-pyrrolin-4-one, potassium salt (proxyl fluorescamine), and 4-((9-acridinecarbonyl)amino)-2,2,6,6-tetramethylpiperidin-1-oxyl (tempo-9-ac).

**APF **is a non-fluorescent molecule until it is reacted with either hydroxyl radicals, peroxynitrite anions (ONOO^-^) [29] or peroxy radicals [35], resulting in cleavage of the aminophenyl ring from the fluorescein ring system, which is highly fluorescent. APF will also be transformed into the fluorescent form if exposed to a combination of H_2_O_2 _and horseradish peroxidase (HRP); HRP catalyzes the oxidation of APF by H_2_O_2_. In a solution containing both H_2_O_2 _and ^•^OH, only ^•^OH will react with the APF unless HRP is added. APF has been used for detecting tobacco-induced intracellular peroxynitrite [36] and we have successfully used APF for detecting ^•^OH generated in pyrite and coal aqueous suspensions [37].

**Amplex ultrared**, an improved version of amplex red (10-acetyl-3,7-dihydroxyphenoxazine), is non-fluorescent until it is reacted with a combination of H_2_O_2 _and HRP. Amplex red is a colorless derivative of dihydroresorufin, which upon oxidation by H_2_O_2 _in the presence of HRP, produces the fluorescent resorufin (3H-phenoxazin-3-one, 7-hydroxy) [38]. Amplex red or amplex ultra red have been used to detect H_2_O_2 _produced in many biological and cell-free systems including glucosides [39] and mitochondria [40], solutions containing ferrous iron [41], mammalian cells exposed to crocidolite asbestos [42], and cigarette smoke that has been bubbled through water [43].

When the diacetate form of dichlorofluorescein, H_2_DCFDA diffuses across cell membranes, it is hydrolyzed by intracellular esterases to form **DCFH **which, upon oxidation by Fenton reaction-generated ^•^OH, H_2_O_2 _& HRP, Fe(II), or peroxynitrite (ONOO^-^), forms the highly fluorescent dichlorofluorescein (DCF) [44]. DCFH has been used to detect silica-induced ROS [45], ambient air particulate-induced ROS in macrophages [46], and welding fume-induced ROS in epithelial cells [47].

**Proxyl fluorescamine **is a radical molecule that was developed by Blough's laboratory [18] to serve as a fluorogenic derivative of the proxyl spin trap: the fluorophore is quenched in the presence of the nitroxide moiety with its delocalized unpaired electron but emits fluorescence when the nitroxide is converted to the corresponding spin-paired hydroxylamine by alkyl radicals. However, proxyl fluorescamine fails to undergo direct reaction with hydroxyl radicals. Blough et al. [48,49] have overcome this lack of reactivity by adding dimethylsulfoxide (DMSO) [< 1 M, which is compatible with normal cell functions (5% DMSO = 705 mM)] to the components of the Fenton reaction [i.e., H_2_O_2 _and Fe(II)]. Under these conditions, the hydroxyl radicals react with the DMSO to form methyl radicals, which then react with proxyl fluorescamine to yield the fluorescent hydroxylamine derivative of fluorescamine. Proxyl fluorescamine has been used to detect ROS generated by suspended diesel exhaust particles [50] and by illuminated retinal membranes [51].

Like proxyl fluorescamine, **tempo-9-ac **is a radical species that does not directly react with hydroxyl radicals. Hydroxyl radicals, however, can convert the probe into a fluorescent product indirectly by attacking other molecules and generating carbon-centered radicals or thiyl radicals that efficiently oxidize tempo-9-ac, converting it to the fluorescent product, 4-[(9-acridinecarbonyl)amino]-2,2,6,6-tetramethylpiperidine (acridine-piperidine) [17]. Borisenko et al. added phenol and hydrogen peroxide to cells and measured the production of ROS. In their system, the peroxidase-catalyzed oxidation of phenol by H_2_O_2 _generated phenoxyl radicals that reacted with intracellular glutathione to form glutathione radicals, which can then directly oxidize the nitroxide and relieve quenching of the acridine ring system. Tempo-9-ac in the presence of exogenous glutathione and phenol can also be used to detect hydroxyl radicals formed from mineral suspensions. Tempo-9-ac has been used to detect ROS generated by photolyzed quinones in solution [52], by vascular smooth muscle cells [53] and ROS associated with hyperglycemia in umbilical vein endothelial cells [54].

### Research strategy

Several probes that fluoresce when reacted with ROS were evaluated for their sensitivity to detect the presence of hydroxyl radicals generated by ferrous iron in solution or in pyrite particle suspensions. In solution, certain transition metals may generate ^•^OH by redox reactions. The reaction of ferrous iron with dissolved molecular oxygen is an example. In these reactions (eqs 1 and 2), ferrous iron reacts with molecular oxygen to form hydrogen peroxide. This hydrogen peroxide can then react with ferrous iron to form hydroxyl radicals through the Fenton reaction (eq. 3).

Fe(II) + O_2 _→ Fe(III) + (O_2_^•^)^-^

Fe(II) + (O_2_^•^)^- ^+ 2H^+ ^→ Fe(III) + H_2_O_2_

Fe(II) + H_2_O_2 _→ ^•^OH + OH^- ^+ Fe(III)

Pyrite (FeS_2_), a mineral commonly found in mining waste, has been previously shown to form ^•^OH [55,21,37]. The mechanism of pyrite-generated ^•^OH is still not completely understood, however ferrous iron that is associated with pyrite may react with molecular oxygen to form hydroxyl radicals (eqs 1 to 3). Considering our previous experience using pyrite [1,21,37,55-60], we chose to use this particle type as a model ^•^OH-generating test particle.

All of the probes were calibrated so that the increase in probe fluorescence after incubation with ^•^OH could be expressed as concentration level of hydroxyl radicals. Hydroxyl radicals can be produced in solution by adding Fenton reagents [i.e., H_2_O_2 _and Fe(II)]; however, predicting the concentration of hydroxyl radicals that are produced in a Fenton reaction is exceedingly difficult. Even if all of the H_2_O_2 _were to stoichiometrically form ^•^OH, any remaining Fe(II) may form additional H_2_O_2 _through other reactions (eqs 1 to 2). Instead of adding Fenton reagents to the probes, a known concentration of hydrogen peroxide was added in the presence of horseradish peroxidase (HRP). Peroxidases catalyze the oxidation of an organic substrate by facilitating electron transfer with hydrogen peroxide. Even in the presence of a peroxidase, however, proxyl fluorescamine and tempo-9-ac will not react efficiently with H_2_O_2_. As indicated above, a carbon radical, derived from exogenously added dimethyl sulfoxide, or a thiyl radical derived from exogenous or endogenous intracellular glutathione, are required to convert proxyl fluorescamine or tempo-9-ac respectively to their fluorescent products.

## Results

**Figure 1:**
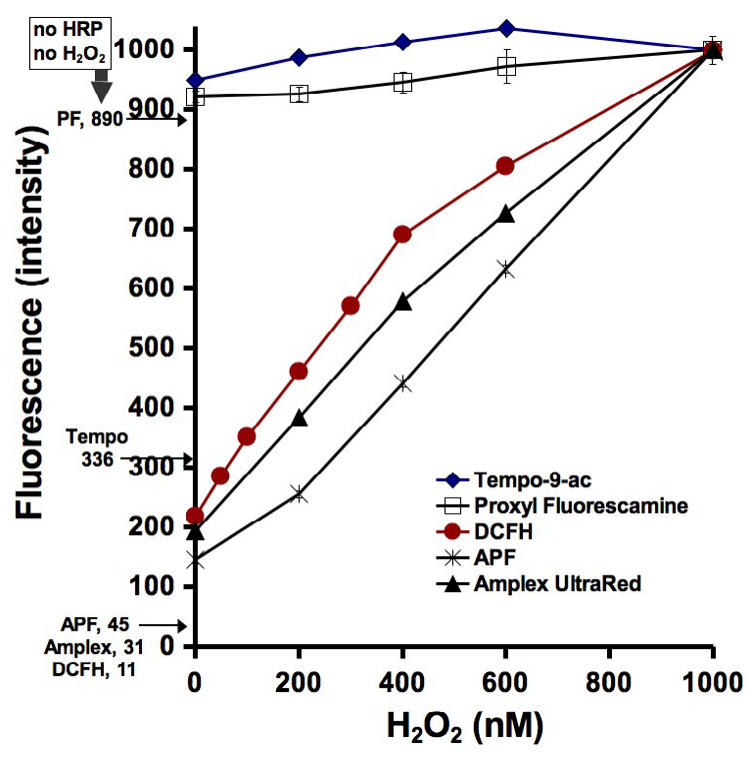
**The effect of H_2_O_2 _concentration on inducing fluorescence in the probes.** In the presence of 0.2 μM HRP, 50 mM phosphate pH 7.4 buffer and H_2_O_2_, oxidation of the probes results in an increase in fluorescence. On the left side of the vertical axis are the fluorescence intensities when no HRP nor H_2_O_2 _are added to the probe and pH buffer. The fluorometer was calibrated so that pure water resulted in a zero fluorescence and the reaction involving the probe with HRP, pH buffer, and 100 nM H_2_O_2 _resulted in a fluorescence reading of 1000. The fluorescence intensity results presented in the next two figures are calibrated using two points: the fluorescence intensity of the probe without H_2_O_2 _or HRP added, and the fluorescence intensity at 1000 nM H_2_O_2_. The measurements were repeated four times and standard deviation error bars have been added. Many of the error bars, however, are within the size of the points.

In order to determine hydroxyl radical generation from hydrogen peroxide in the presence of HRP or from ferrous iron solutions and particulate suspensions, calibration curves were first produced. In the presence of HRP, the fluorescence intensity of all of the probes increases with increasing hydrogen peroxide concentration (Fig. 1). The gain on the fluorimeter was adjusted in this series of experiments to display a relative fluoresence intensity of 1000 in the presence of 1000 nM H_2_O_2 _along with 0.2 μM HRP and each probe at its concentration as listed above in **Methods**. The baseline of the instrument was adjusted to display a relative intensity of zero in the presence of water alone. It is evident from Figure 1 that in the absence of H_2_O_2_, all of the probes except for proxyl fluorescamine and tempo-9-ac show relative fluorescence intensities that are less than 5% of the signals they generate in the presence of 1000 nM H_2_O_2 _and HRP. With addition of HRP alone, in the absence of exogenously added H_2_O_2_, APF, DCFH, and amplex ultrared show small increases in fluorescence intensity which may reflect spontaneous formation of H_2_O_2 _but these signals are still less than 20% of the signals generated in the presence of 1000 nM exogenous H_2_O_2 _and HRP. The two fluorogenic free radical scavengers, tempo-9-ac and proxyl fluorescamine, however, clearly undergo significantly smaller increases in fluorescence with increasing H_2_O_2 _concentration than the other probes. We hypothesize that these smaller increases reflect the indirect mechanisms by which hydroxyl radicals modify the two spin traps (through phenoxy- and thiyl radicals in the case of tempo-9-ac and through methyl radicals in the case of proxyl fluorescamine), resulting in suboptimal conversion of the probes to the spin-neutral species.

In order to measure hydroxyl radicals generated by ferrous iron solutions and pyrite suspensions, a different calibration curve was used. The gain was still adjusted to display a relative fluorescence intensity of 1000 in the presence of 1000 nM H_2_O_2 _and HRP, but the signal from each probe in the absence of H_2_O_2 _and HRP (i.e., values to the left of the vertical axis), rather than the signal from water alone, was set to a relative fluorescence intensity of zero.

**Figure 2:**
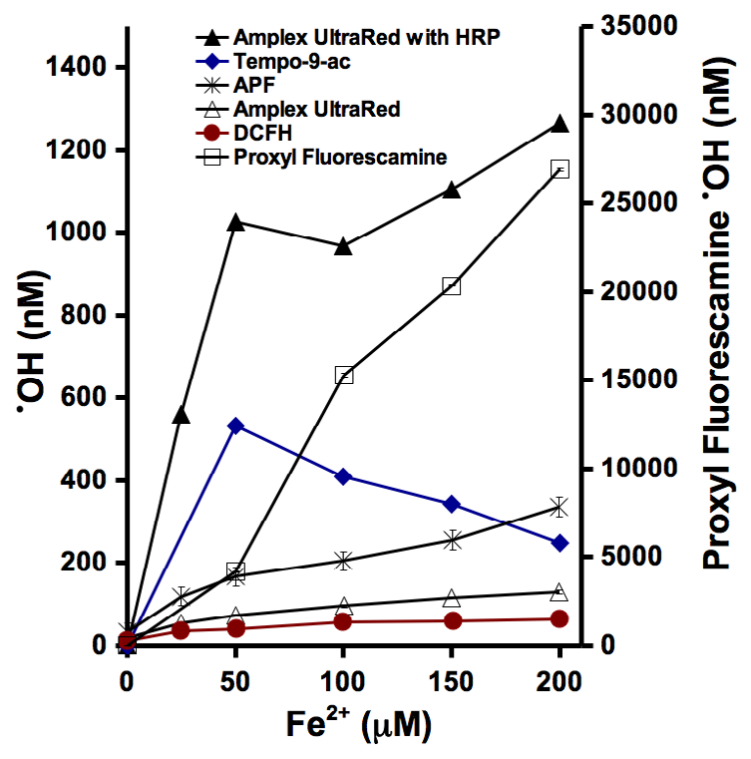
**Iron-induced formation of reactive oxygen species.** In the presence of water and dissolved oxygen, ferrous iron will form both H_2_O_2 _and hydroxyl radicals (eqs 1 to 3).

In an aqueous solution containing dissolved oxygen, the addition of soluble ferrous salts or ferrous iron-containing minerals such as pyrite results in the formation of both hydrogen peroxide and hydroxyl radicals (eqs 1 and 2). All of the probes were incubated with multiple concentrations of ferrous iron in 25 mM phosphate buffer, pH 7.4 (Fig. 2). The results show differences in each probe's sensitivity to hydroxyl radicals generated in ferrous iron solutions. When less than 50 μM ferrous iron salts were added to the probes, the fluorescence intensities of all of the probes were observed to increase. The apparent concentration of detected hydroxyl radicals declined when greater concentrations of ferrous iron were added to tempo-9-ac. Since the calibration curve generated for proxyl fluorescamine has a very shallow slope, small changes in the fluorescence intensity of this probe equate to very large apparent changes in hydroxyl radical concentration, reflected in the vertical axis for proxyl fluorescamine on the right side of Figure 2. An additional complication in detection of hydroxyl radicals generated by ferrous salts and minerals using amplex ultrared as a probe arises from the capacity of the dye to be oxidized by H_2_O_2 _to the fluorescent resorufin in the presence of HRP. It is evident that H_2_O_2 _is indeed formed in addition to hydroxyl radicals upon addition of ferrous iron salts or minerals because addition of HRP to the incubation mixtures containing the iron samples and amplex ultrared results in a significant increase in fluorescence.

**Figure 3:**
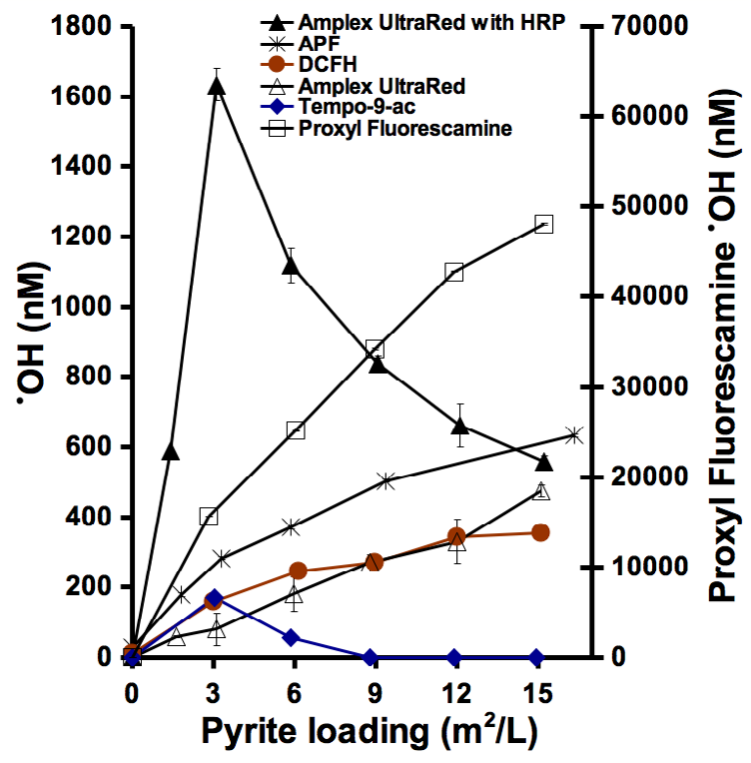
**Pyrite-induced formation of reactive oxygen species.** Varying amounts of pyrite particles were incubated with the probes for 24 hrs followed by filtration and fluorescence measurements.

One of our goals of having an ROS detection method is for its implementation for the detection of particulate-generated hydroxyl radicals. From previous research, we know that aqueous suspensions of pyrite particles will form hydroxyl radicals [21]. In this study, all of the probes were incubated with several different concentrations of pyrite particles and then evaluated for the generation of hydroxyl radicals (Fig. 3). The results show that for all of the probes except amplex ultrared (in the presence of HRP) and tempo-9-ac, fluorescence intensity generated by the probes increases proportionally to the total pyrite surface area exposed to the aqueous solvent. In other words, the probes record an increase in the formation of hydroxyl radicals with an increase in available surface area. The increased fluorescence from tempo-9-ac in the presence of pyrite particles shows a similar iron dose dependence to that observed in the presence of soluble ferrous salts: the fluorescence increases with small amounts of ferrous iron or pyrite and then decreases when more of the iron-containing species is added. The results from amplex ultrared are similar but when no HRP is added, the fluorescence increases nearly linearly. As in the studies with soluble ferrous iron salts described above, the sensitivity of proxyl fluorescamine to hydroxyl radicals from pyrite suspensions is so low that the concentrations of hydroxyl radicals formed cannot be detected with precision, and the apparent values, shown on the right vertical axis, are clearly outside the range of those detected by the other probes.

## Discussion

A major goal of these studies was to evaluate several fluorogenic probes for their sensitivity to hydroxyl radicals, especially at the lower limit of detection, as well as their suitability for use with particulate mineral suspensions. Specifically, we have used the probes to determine the apparent concentrations of hydroxyl radicals generated in a 24 hour time period, at room temperature, in phosphate buffer at pH 7.4. Changing any of these variables would probably lead to different apparent concentrations of hydroxyl radicals being detected. However, by keeping these variables constant, a comparison of all the probes could be performed for three systems: (1) H_2_O_2 _and HRP, (2) Fe(II), and (3) pyrite suspensions.

After reaction with H_2_O_2 _and HRP, each probes fluorescence' is different (Fig. 1). The curves from DCFH, APF, and amplex ultrared are similar but different from those recorded for tempo-9-ac and proxyl fluorescamine. This is expected because the former three will directly react with H_2_O_2 _in the presence of HRP and hence their greater sensitivity (APF is the most sensitive of all probes). Because both tempo-9-ac and proxyl fluorescamine will not directly react with H_2_O_2 _in the presence of HRP, their responsiveness' is reduced. In the presence of HRP but absence of H_2_O_2_, all of the probes exhibit some level of fluorescence. This fluorescence level is higher than the background fluorescence level for each probe (listed on the left side of the vertical axis, Fig. 1). This indicates that either there is some H_2_O_2 _in the water that was used to dilute the solutions in the experiments, or that HRP directly reacts with the probes.

Calibrating the fluorometer is necessary but it presents some challenges.

The fluorometer was calibrated with water as zero fluorescence and a reaction of H_2_O_2_/HRP/glutathione & phenol (for tempo-9-c)/DMSO (for proxyl fluorescamine) as a fluorescence value of 1000. Compared to the fluorescence of only DCFH, APF, and amplex ultrared in a pH buffered water solution, the addition of H_2_O_2 _& HRP results in much greater fluorescence intensity. When the fluorometer is calibrated with this large difference in fluorescence intensities, subsequent measurements have a greater accuracy. Both tempo-9-ac and proxyl fluorescamine do not respond with as great of an increase in fluorescence when incubated with H_2_O_2 _and HRP. Therefore, the sensitivity of tempo-9-ac and proxyl fluorescamine are lower than the sensitivities of DCFH, APF, and amplex ultrared. The background fluorescence for proxyl fluorescamine is only slightly less than the fluorescence of proxyl fluorescamine reacted with 1 μM H_2_O_2 _and HRP (i.e., 890 compared to 1000). When proxyl fluorescamine is incubated with either Fe(II) (Fig. 2) or pyrite (Fig. 3), the larger fluorescence intensities measured are calculated as significant concentrations of hydroxyl radicals. This problem may be resolved by using more than 1 μM H_2_O_2 _in the calibration of proxyl fluorescamine, but that would defeat our objective of comparing all of the probes uniformly.

If the probes reacted equally with a ferrous iron solution, then they would all detect the same concentration of hydroxyl radicals and all of the curves would overlap. The results shown in Figure 2 show different calculated concentrations of hydroxyl radicals being detected. The trend of tempo-9-ac is unique because it's fluorescence increases with Fe(II) up to 50 μM, but then the fluorescence decreases when the concentration of Fe(II) is further increased (Fig. 2). The same result occurs when tempo-9-ac is incubated with pyrite particles (Fig. 3). The increasing fluorescence intensities of the other probes with increasing loadings of pyrite and Fe(II), suggests that tempo-9-ac is either only capable of detecting small concentrations of hydroxyl radicals or another factor is limiting it's fluorescence such as an interaction between iron and the probe. The addition of HRP to amplex ultrared results in a greater detected concentration of ROS. A ferrous iron solution will generate both hydrogen peroxide and hydroxyl radicals (eqs 1 to 3). In the presence of HRP, the relative contribution to amplex ultrared fluorescence' due to hydroxyl radicals versus hydrogen peroxide remains unknown.

In the presence of pyrite particles (Fig. 3), each probe's increase in fluorescence shows similar trends to the results when the probes are incubated with ferrous iron (Fig. 2). The main differences are in the reactivities of amplex ultrared with HRP and tempo-9-ac. For these probes, increasing the pyrite loading above 3 m^2^/L results in a decrease in the detected hydroxyl radical concentration. This may be due to several factors: (1) a decrease in the stability of amplex ultrared in the presence of greater concentrations of hydroxyl radicals, (2) amplex ultrared adsorption to the larger surface areas of pyrite present in higher loadings, (3) an interaction between iron and the probe or one of the reactants (e.g., glutathione), or (4) alteration of solution pH in the presence of higher loadings of pyrite. Although we evaluated pyrite particles for generation of ROS for 24 hours, the time span of incubation with the probes could have been shorter or longer. The probes appear to increase in fluorescence over time when incubated with particles suggesting that the detection of ROS is cumulative.

Throughout the study, the solutions containing APF were the easiest to prepare, contained a minimal amount of additional reactants and the results were the easiest to interpret. Unlike DCFH, APF was used directly from the purchased vial without further treatment. Both proxyl fluorescamine and tempo-9-ac required addition of DMSO and glutathione/phenol, respectively. Proxyl fluorescamein and tempo-9-ac also required preparation from powders and quantification using UV-Vis spectroscopy – APF was purchased as a 5 mM solution.

## Conclusion

The objective of these studies was to evaluate several fluorescence-based techniques for measuring hydroxyl radicals in solution with the ultimate goal of using one or more of the techniques to compare the concentration of hydroxyl radicals that are generated in particulate suspensions. The results show that some probes are more suitable for these goals. APF, DCFH, and amplex ultrared do not require extra reactants other than water and a pH buffer to quantify hydroxyl radicals, which results in their higher sensitivity and ease of use. Proxyl fluorescamine and tempo-9-ac both require other reactants and their use is limited to high precision measurements and solutions with low concentrations of reactants such as ferrous iron and pyrite, respectively. DCFH and amplex ultrared would also be suitable but DCFH requires an additional de-esterification step for its use in cell-free solutions and both DCFH and amplex ultrared will react with ROS other than hydroxyl radicals. APF is both sensitive and selective for only the most highly reactive ROS, including hydroxyl radicals. The fluorogenic probes that were evaluated here were chosen because they can be purchased and used with little further treatment. This study is not an extensive study of all methods available and many other probes are available for the detection of ROS; for reviews, see [61-63].

## Methods

### ^•^OH measurement

The methods for measurement of hydroxyl radicals using the different probes were all performed in a similar fashion. All of the probes were purchased from Invitrogen and, except for H_2_DCFDA, were used without further treatment. In all of the experiments, the reactant solutions, including mineral particles if used, were combined in 2-mL centrifuge tubes at room temperature (22 ± 2°C). The tubes were placed on an end-over-end orbital mixer in the dark to prevent photo-activated oxidation. After 24 hours, particulate suspensions were filtered through Millipore PVDF 0.45 micron filters and, subsequently, transferred to 4-mL methylcrylate fluorescence cuvettes prior to fluorescence measurements using the appropriate wavelength filters. The filtration step was omitted in experiments conducted without particles.

Calibration curves were generated similarly for all of the fluorescent probes. A known amount of H_2_O_2 _was added to the probe dissolved in 50 mM potassium phosphate buffer at pH 7.40, along with 2.95 units/mL (equivalent to 0.2 μM) Sigma type II horseradish peroxidase (HRP). The reaction between H_2_O_2 _and horseradish peroxidase oxidizes the probes stoichiometrically, resulting in an increase in probe fluorescence. This method for oxidizing the probe molecules was used instead of employing the Fenton reaction to generate ^•^OH because of the difficulty in controlling the quantity of ^•^OH formed in the Fenton reaction. By using a series of calibration curves generated from known quantities of H_2_O_2 _in the presence of HRP, the values of relative fluorescence intensity from probes in the experiments described below could all be converted to corresponding concentrations of ^•^OH (nM). The water used in the experiments (Easy Pure 18.3 MΩ-cm, UV-irradiated, ultrafiltered) was stored in the dark for at least one month before being used in experiments to limit the background concentration of H_2_O_2_.

### Aminophenyl fluorescein (APF)

3'-(*p*-aminophenyl) fluorescein (APF) is not fluorescent until reacted with ^•^OH [29] or with H_2_O_2 _in the presence of horseradish peroxidase (HRP). 10 μM APF was employed in these studies; its cleavage to fluorescein was quantitated using a Tuner Barnstead spectrofluorimeter with excitation and emission wavelengths set to 490 nm and 520 nm, respectively.

### Amplex ultrared

10-acetyl-3,7-dihydroxyphenoxazine (amplex ultrared) will react with H_2_O_2 _in the presence of HRP to produce highly fluorescent resorufin (3H-Phenoxazin-3-one, 7-hydroxy) [38]. In these studies we employed 50 μM amplex ultrared in the presence and absence of 2.95 units/mL (equivalent to 0.2 μM) HRP to detect ROS. The formation of resorufin was quantitated spectrofluorimetrically with excitation and emission wavelengths of 540 nm and 590 nm, respectively.

### Dichlorodihydrofluorescein (H_2_DCFDA)

2',7'-dichlorodihydrofluorescein diacetate (H_2_DCFDA) will become oxidized to the fluorescent 2',7'-dichlorofluorescein (DCF) in the presence of hydroxyl radicals, HRP & H_2_O_2_, peroxynitrite, or nitric oxide [30,31]. In order for H_2_DCFDA to be converted to DCF, it must first be de-esterified, which is achieved by nonspecific esterases in the cytosol of viable cells but which is accomplished by acid- or base-catalyzed esterolysis in the absence of cells [30]. Here, we employed an alkaline hydrolysis protocol described by LeBel et al. [30]: (a) 0.0049 g of H_2_DCFDA was dissolved in 10 mL methanol, (b) 500 μL of this 1 mM H_2_DCFDA solution was added to 2 mL of 0.01 N NaOH, (c) after 30 minutes, 10 mL of a 25 mM phosphate buffer at pH 7.4 was added to the H_2_DCFDA/methanol/NaOH solution. In the experiments, 50 μL of this solution was added to the reaction solutions so that the final concentration of H_2_DCFDA was 10 μM. The formation of DCF was quantitated spectrofluorimetrically with excitation and emission wavelengths of 490 nm and 520 nm, respectively.

### Proxyl fluorescamine

A stock solution of 5-(2-carboxyphenyl)-5-hydroxy-1-[(2,2,5,5-tetramethyl-1-oxypyrrolidin-3-yl)methyl]-3-phenyl-2-pyrrolin-4-one, potassium salt (proxyl fluorescamine) was prepared by dissolving 5 mg of proxyl fluorescamine in 5.125 mL DMSO to obtain a 2 mM proxyl fluorescamine solution. An aliquot of the stock solution was added to reaction vials containing a total volume of 1.7 mL in which components for generation of hydroxyl radicals were present so that the final concentration of proxyl fluorescamine was 100 μM and 5% DMSO was present. Fluorescamine fluorescence was quantitated spectrofluorimetrically with excitation and emission wavelengths of 390 nm and 490 nm, respectively.

### Tempo-9-ac

4-((9-acridinecarbonyl)amino)-2,2,6,6-tetramethylpiperidin-1-oxyl (tempo-9-ac) has been developed as a fluorogenic spin trap by Borisenko et al. [17] to serve as an alternative to proxyl fluorescamine. A solution of tempo-9-ac was prepared by dissolving powdered tempo-9-ac in DMSO and determining its concentration from its absorbance at 359 nm (ε = 10.4 mM^-1 ^[17]). All of the experiments with tempo-9-ac were carried out in the presence of 100 μM tempo-9-ac, 1 mM glutathione, and 100 μM phenol in 25 mM phosphate buffer pH 7.4. Acridine fluorescence was measured spectrofluorimetrically with excitation and emission wavelengths of 360 nm and 420 nm, respectively.

### Mineral sample preparation

Natural pyrite (Huanzala, Peru) obtained from Ward's Natural Science, Rochester, NY was crushed in an agate mill and was sieved to obtain particle sizes between 38 to 63 μm. The ground pyrite was stored in a vacuum desiccator until used in the experiments. A surface area of roughly 1.25 m^2^/g was determined using five-point N_2 _adsorption BET.

## Authors' contributions

CAC designed the study, performed the experiments and drafted the manuscript. SRS participated in the design of the study. MAAS coordinated the study and provided funding. All authors read and approved the final manuscript.
